# Effects of Fabric Counts and Weave Designs on the Properties of Laminated Woven Kenaf/Carbon Fibre Reinforced Epoxy Hybrid Composites

**DOI:** 10.3390/polym10121320

**Published:** 2018-11-28

**Authors:** H.A. Aisyah, M.T. Paridah, A. Khalina, S.M. Sapuan, M.S. Wahab, O.B. Berkalp, C.H. Lee, S.H. Lee

**Affiliations:** 1 Institute of Tropical Forestry and Forest Products (INTROP), Universiti Putra Malaysia, 43400 UPM Serdang, Selangor, Malaysia; a.humaira.aisyah@gmail.com (H.A.A.); khalina@upm.edu.my (A.K.); sapuan@upm.edu.my (S.M.S.); leechinghao@upm.edu.my (C.H.L.); 2 Faculty of Engineering, Universiti Putra Malaysia, 43400 UPM Serdang, Selangor, Malaysia; 3 Faculty of Mechanical Engineering, Universiti Tun Hussien Onn Malaysia (UTHM), 86400 Batu Pahat, Johor, Malaysia; saidin@uthm.edu.my; 4 Faculty of Textile Technology and Design, Istanbul Technical University, İnönü Caddesi. No.: 65, Gumussuyu, Beyoglu, 34437 Istanbul, Turkey; berkalp@itu.edu.tr

**Keywords:** fabric, woven kenaf, plain, satin, hybrid composites

## Abstract

The effects of different fabric materials namely weave designs (plain and satin) and fabric counts (5 × 5 and 6 × 6) on the properties of laminated woven kenaf/carbon fibre reinforced epoxy hybrid composites were evaluated. The hybrid composites were fabricated from two types of fabric, i.e., woven kenaf that was made from a yarn of 500tex and carbon fibre, by using vacuum infusion technique and epoxy resin as matrix. The panels were tested for tensile, flexural, and impact strengths. The results have revealed that plain fabric is more suitable than satin fabric for obtaining high tensile and impact strengths. Using a fabric count of 5 × 5 has generated composites that are significantly higher in flexural modulus as compared to 6 × 6 which may be attributed to their structure and design. The scanned electron micrographs of the fractured surfaces of the composites demonstrated that plain woven fabric composites had better adhesion properties than satin woven fabric composites, as indicated by the presence of notably lower amount of fibre pull out.

## 1. Introduction

Composite materials with natural fibre as a means of reinforcement are becoming increasingly prevalent in many applications such as semi-structural building component, automotive, furniture, and other applications. The most significant criteria of natural fibres are renewability, bio-degradability, light weight, good mechanical properties, and having low density with good strength. On the other hand, substitution of conventional materials with natural fibres textiles as a reinforcement agent prevails all over the world as they offer several advantages including high strength and stiffness, are durable and have superior design flexibility [1,2]. Many researchers have studied the utilisation of natural fibres as textile reinforcement with polymer matrix such as those from hemp [3,4], jute [5,6,7,8], flax [9,10], and kenaf [11,12,13,14,15]. Over the years, kenaf fibre has been utilised extensively as a reinforcement agent in many productions of polymer composites. Low density and high specific mechanical properties of kenaf have become a pulling factor for kenaf as a preferred natural reinforcement fibre in the biocomposite industry. Akil et al. [16] has stated that kenaf fibre reinforced polymer composite, and thus has good potential among other natural fibres due to its excellent properties. 

Woven materials are formed by interlacing vertical yarn (warp) and horizontal yarn (weft) to form a fabric. Performance of woven composite is governed mainly by the textile/fabric properties. According to Das [17], the main elements and critical factors that control the fabric properties are yarn properties, fabric count, and weave design. The manipulation of these elements would produce fabric with different physical and mechanical properties. For instance, Alavudeen et al. [18] has found that the plain fabric of banana/kenaf reinforces polyester composite and shows improved tensile properties compare to the twill fabric weave design. A study by Wahab and co-workers [19] on woven composite have shown that the performance of woven kenaf composite are affected by yarn size and weave design that determine the woven fabric porosity and crimp percentage. These are the main factors that control the mechanical properties of the composite. Furthermore, the addition of woven material improves the fracture toughness of the flax composites as has been reported by Liu and Hughes [20].

Numerous researches have been done by utilising natural fibre in a form of fabric to produce woven composite (Table 1). Saiman et al. [21] have fabricated a series of woven kenaf composite with different yarn linear densities and weave structures using a polyester (PE) matrix through a vacuum infusion technique and have found that both parameters have a significant influence on the tensile properties of the composite. Research that has been done by Azrin Hani et al. [12] have demonstrated that woven kenaf and coir have a high potential to be used as reinforcing materials, and they also concluded that fibre type and reinforcement structure parameters affect the mechanical properties of the composites. Unfortunately, the mechanical properties of natural fibres composite do not match up to those of synthetic fibre composite. One solution to this problem is to replace only a fraction of the synthetic fibres, thus making a hybrid composite. Hybridisation of synthetic fibre with natural fibre has many benefits such as excellent thermal and mechanical properties.

In order to optimise the performance of the woven composite from natural fibres, hybridisation with synthetic fibre is believed to improve the plant based material for composite due to its comparable composite performance for several applications. According to Jambari et al. [26], hybridisation in composite area is a method of the combination of different resources and processes with different properties for the improvement of existing material. Several studies have been carried out on utilising kenaf yarn for fabric composite production [18,19,21,24,27]. The idea of hybrid weaving between synthetic fibres and kenaf fibres is highly recommended to compensate for the dramatic loss of strength. One study has found that pure woven kenaf and pure woven Kevlar epoxy composite has a tensile value of 16MPa and more than 250 MPa, respectively. The hybridisation affected the intermediate mechanical properties compared to the highest Kelvar/epoxy properties and the lowest properties of kenaf/epoxy composite [28].

A number of studies have reported on the hybridisation of natural fibres with carbon fibre to produce a woven hybrid composite for several applications [29,30,31]. In these studies, reinforcement is by adding one or more layers of natural fibres in an interlaminate of carbon fibre composite that would lead to a formation of a hybrid composite with a great diversity of material properties. Furthermore, alteration of fabric properties such as weave design and the fabric count give flexibility to tailor the final composite properties according to the requirements, which is one of the major advantages of the textile composites. Thus, in this study, fabrics from kenaf fibre and carbon fibre were used to fabricate a three-layered laminated woven hybrid composite, where woven kenaf is the core layer and carbon fibres is the top and bottom layers. The kenaf woven fabric was made from different fabric counts and weave designs as these parameters would have some effects on the performance of the hybrid composite. This article reports on the physical, mechanical (tensile, flexural, and impact strengths), and morphological properties of laminated woven kenaf/carbon fibre reinforced epoxy hybrid composites. 

## 2. Materials and Methods

### 2.1. Materials

**Figure 1:**
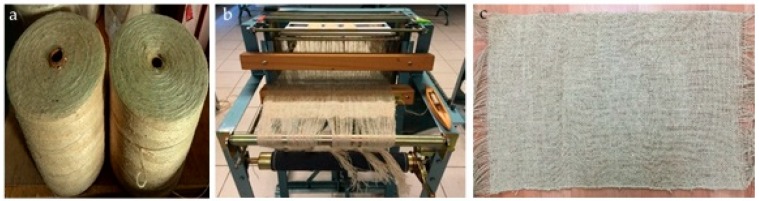
(**a**) Kenaf yarn with 500tex linear density wound on cylindrical bobbins; (**b**) hand loom used in the weaving process; (**c**) kenaf woven fabric.

The materials used in this study were woven kenaf, carbon fibre fabrics and epoxy resin. Kenaf yarn with 500tex (Figure 1a) was weaved using a hand loom machine (Gulas Makine, Fatih, Istanbul, Turkey) (Figure 1b) to produce woven fabric (Figure 1c), with two weaving designs, plain and satin structure. The plain structure is the simplest weave where the yarn interlaces in alternate order. Satin structure was weaved by four of the weft yarns floating over a warp yarn or four warp yarns floating over a single weft yarn. Two different fabric counts were selected, they were 5 × 5 and 6 × 6 fabric (number of warp yarn × number of weft yarn). 

**Figure 2:**
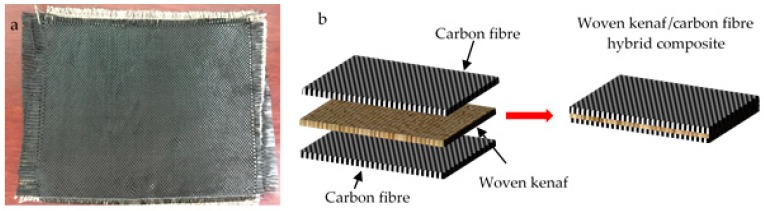
(**a**) Carbon fibre and (**b**) Three-layered woven hybrid composite sample prepared using vacuum infusion process.

Carbon fibre (Figure 2a) in a fabric form with a plain woven structure was supplied by Spinteks Tekstil Ins. (Honaz, Denizli, Turkey). Specific properties of the carbon fibre were tabulated in Table 2. The matrix used to fabricate the samples was EPIKOTE Resin 240 (Chemrex Corporation, Cheras, Selangor, Malaysia), industrial liquid epoxy resin with a density of 1.12 g/cm^3^ and hardener EPIKURE Curing Agent 3090 supplied by Chemrex Corporation Sdn. (Cheras, Selangor, Malaysia).

### 2.2. Fabrication of Woven Kenaf/Carbon Fibre Hybrid Composite

The composite was prepared through vacuum infusion in order to efficiently pull the epoxy resin into the layer of woven kenaf and carbon fibre by removing the air from the system. Each composite consists of a single ply woven kenaf as the reinforcement at the centre, and carbon fibres at the upper and lower layers (Figure 2b). The compositions of laminated hybrid composites are shown in Table 3. The ratio of woven kenaf-carbon fibre and epoxy resin was 30:70 by mass. The samples were prepared by hand lay-up method followed by vacuum bagging. The release agent was applied to the surface of a glass mould to ease the sample removal once cured. Then, the vacuum pump was switched on until the infused materials are compact. The epoxy was infused from the resin tank until excess resin flowed into the resin trap vessel. The resin was then allowed to flow for a few minutes to ensure that the resin penetrated all the layers. Finally, the infused fabric composite was left to cure for 24 h at room temperature. The carbon fibre epoxy specimens were also prepared as the control sample.

### 2.3. Evaluation of Composite Properties

All samples were tested for physical (fibre volume fraction, density, and void contents) and mechanical properties (tensile test, flexural test, and impact test) according to the ASTM Standard. The density of the woven kenaf, carbon fibre and woven hybrid composite were measured based on ASTM D3800-99. The samples were conditioned for 24 h, and the density of both fibres was measured using the Mettler Toledo (XS205) density kit (Columbus, OH, USA). The average density of woven kenaf (kf), carbon fibre (cf) and composite of ten specimens were taken and recorded. 

The volume fractions of composite (V_f_) was calculated by using Equation (1). The fibre volume fraction for woven kenaf (kf) and carbon fibre (cf) were calculated based on Equations (2) and (3), respectively.

V_f_ = (W/*ρ*)_fibre_/((W/*ρ*)_fibre_ + (W/*ρ*)_epoxy_) (1)

V_kf_ = (W/*ρ*)_kenaf_/((W/*ρ*)_kenaf_ + (W/*ρ*)_carbon_ + (W/*ρ*)_epoxy_) (2)

V_cf_ = (W/*ρ*)_carbon_/((W/*ρ*)_carbon_ + (W/*ρ*)_kenaf_ + (W/*ρ*)_epoxy_) (3)

where (W/*ρ*) are the known weights and density of woven kenaf, carbon fibre and epoxy resin, respectively. Based on the determined volume fraction of composite, woven kenaf, carbon fibre and matrix, voids (V_v_) in the composites were calculated according to Equation (4).

V_v_ = 1 − (V_kf_ + V_cf_ + V_f_) (4)

Tensile specimens were cut into 250 × 25 mm × actual thickness with gauge length of 170 mm. The test was conducted based on ASTM D3039 using a universal testing machine (Instron P5567, Norwood, MA, USA) at the crosshead speed of 2 mm/min. For each variable, 10 specimens were tested and the average values for tensile strength and tensile modulus were obtained. The rectangular specimens with dimension of 100 × 20 mm were cut and flexural test was conducted through three-point loading using a universal testing machine (Instron P5567) according to ASTM D790 at the crosshead displacement rate of 5 mm/min. Ten specimens were tested for each sample and average values were recorded. Modulus of elasticity of the composites was calculated based on the slope (m) of load–displacement curves using Equation (5).

E = (L²m/4bd²)(5)

where, L, b, and d represent support span, width of sample and depth of samples, respectively.

The impact strength was measured using Charpy impact test machine (Instron Ceast 9050, Norwood, MA, USA) according to the ASTM D6110. Ten specimens for each composite sample were cut into a dimension of 127 × 12.7 mm × actual thickness for the striking hammer energy of 5 J.

### 2.4. Morphological Observation

Hitachi 3400 SEM (Chiyoda, Tokyo, Japan) was used to observe the tensile fracture surfaces of the woven kenaf/carbon fibre hybrid composite laminated composite. The fractured part of the samples was cut and the SEM micrographs were taken to investigate the fractured mechanisms and interface adhesion of the composites. All samples had been sputter-coated with gold with the acceleration voltage at 1 kV to avoid charging.

### 2.5. Data Analysis

The data were statistically analysed using a statistical analysis system (SAS) software (SAS Institute, Cary, NC, USA) that applied analysis of variance (ANOVA) and the least significant difference (LSD) method for mean separation, to evaluate the effects of types of fabric count and weave design on the panel properties. LSD method calculates the least difference that must occur between two means and compare them at *p* ≤ 0.05. Means that differ more than the value is considered significantly different from each other and is ranked as a, b, c, d and e. According to this method, means having the same letters are not significantly different from each other at *p* ≤ 0.05.

## 3. Results and Discussion

### 3.1. Volumetric Composition of Woven Kenaf/Carbon Fibre Hybrid Composite

Table 4 shows the results for the density, fibre volume fraction and the void content of the composite as a function of fabric count and weave design. It can be seen that, the density of the composites varies between samples. CP5 (composite with plain fabric and 5 × 5 fabric count) shows the lowest density of 0.98 g/cm^3^, while CS6 (composite with satin fabric and 6 × 6 fabric count) shows the highest density of 1.24 g/cm^3^, about 26.5% denser than CP5. Obviously, the high variation in the density of the three-layered woven kenaf/carbon fibre hybrid composite is attributed to the fabric areal density. Satin fabric has a higher areal density and thickness than plain fabric owing to its design structure and fabric arrangement. In addition, the fabric count had also contributed to the increase in weight and consequently resulted in density increment. For instance, fabric having fabric count 6 × 6 has a higher density than those made with 5 × 5 since it contains more fibres, i.e., 6 yarns in warp and weft directions, as compared to 5 yarns in warp and weft directions. 

The fibre volume fractions of woven kenaf (V_kf_) were found to be higher than that of the carbon fibre (V_cf_). Fibre volume fraction is the ratio of fibre volume by composite volume and is mostly dependent on the density of the materials that are used in composite production [32]. Composites made from woven kenaf significantly increased its weight fraction and volume fraction as different weave design and different fabric count were used. On the other hand, carbon fibre maintained its weight fraction (11.27–12.0%) and volume fraction (12.23–12.31%) irrespective of weave design or fabric count used. Composite of 6 × 6 of fabric count was found to have higher value in V_f_ than those of 5 × 5. The introduction of fabric count of 6 × 6 increases the V_kf_ by 8 to 10%, which implies that V_f_ is more governed by woven kenaf than carbon fibre. 

The void content of the composite for different types is shown in Table 4. Apparently, CS6 (Satin, 6 × 6 fabric count) gives the highest void content in spite of having the highest density and V_f_. Two plausible explanations for this are: (1) satin fabric has a loose structure compare to plain fabric, thus results in higher void content and (2) 6 × 6 fabric count structure is very tight, thus the flow of the resin is less efficient-creating voids and delaminations between the fibres. According to Goodwin et al. [33], in the satin fabric laminates composites, the number of voids is higher than in plain fabric laminates composites and is reflected in the reduction on shear strength value. 

### 3.2. Mechanical Properties of Woven Kenaf/Carbon Fibre Hybrid Composite

The results of the ANOVA shown in Table 5 suggest that the weave design has highly significant effect on tensile strength (*p* ≤ 0.01) while flexural modulus, impact strength and impact energy were also significantly influenced (*p* ≤ 0.05). On the other hand, fabric count was found did not exert any significant influence on the mechanical properties of woven kenaf/carbon fibre hybrid composite (*p* > 0.1). The ANOVA results also suggest that there are no interaction effects between both fabric count and weave design on all the properties except for flexural strength. Hence the following discussions are based on factors that exert significant effects to the mechanical properties of woven kenaf/carbon fibre hybrid composites.

#### 3.2.1. Tensile Strength

Since the ANOVA showed tensile strength had been significantly affected by the weave design only, a further evaluation on means separation-using least significant difference (LSD) method, was conducted and the results are shown in Table 6. Composites made from satin-designed woven kenaf had a significantly lower tensile strength and modulus by 14.76% and 8.42%, respectively, than plain-designed woven kenaf. Tensile strength is dependent on the weaving factors such as yarn linear density, weave design, fabric density or yarn spacing, and fabric crimp. It is also influenced by the lamination structures (fibre/fabric orientations, fibre volume fraction) as well as the inherent properties of the materials, i.e., fibres and matrix [28,34,35]. This finding was supported by Chow et al. [36] who found that plain woven composite lead to an improvement in tensile strength and modulus, this was mainly attributed to the minimum force development that was caused by the distribution of load transfer along the fibre direction. 

**Figure 3:**
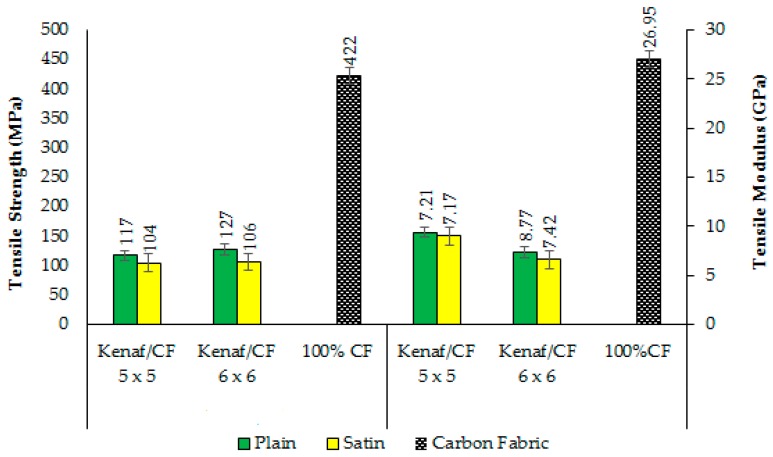
Tensile strength and tensile modulus of woven kenaf/carbon fibre hybrid composite.

Figure 3 compares the tensile strengths of all types of woven kenaf/carbon fibre hybrid composite and carbon fibre composite produced in this study. In general, it can be seen that tensile properties of the plain-designed composite were much higher than those of satin-design composite, irrespective of fabric count (5 × 5 or 6 × 6). This finding was probably due to a more uniform distribution of tensile load transfer in both warp and weft direction in a plain fabric than in satin fabric. The former contains symmetrical fabric structure thus it can provide a much more consistent transfer of stress from one layer to another fibre layer [37].

**Figure 4:**
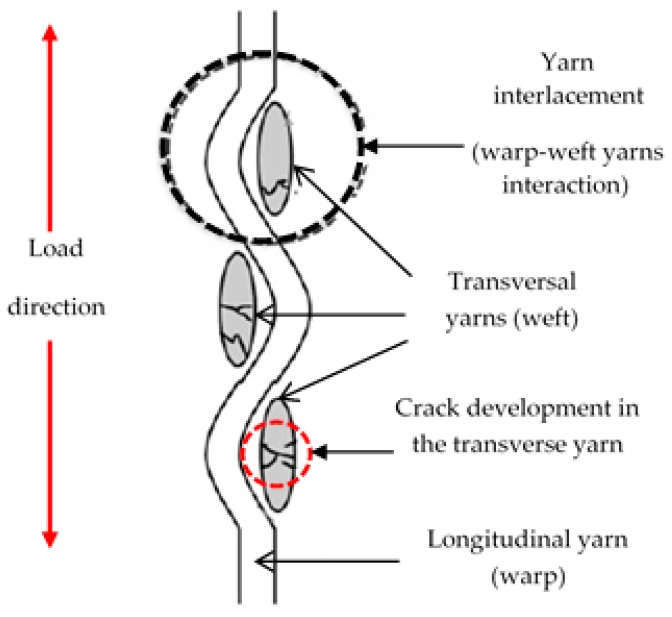
Development of failure perpendicular to the loading direction i.e., in transverse direction [35].

When loaded in tension, i.e., tensile test, the composite experienced fractures in the transverse direction, which normally is associated with extensive longitudinal splitting or failure of the specimens, as shown in Figure 4. Morphological analysis on damaged areas suggests that the transversal cracks are apparently developed perpendicularly to the loading direction, i.e., in the transverse direction [38,39]. The crack promotes further delamination and failure of the specimens. Rios-Soberanis et al. [38] explained that transversal cracks would usually led to small delaminations in the interlacement interface area between yarns. They have also identified that the interlacement of warp-weft yarn is the origin of the cracks that occur due to high stress concentrations, hence acting as crack-initiation points.

This is in agreement with the findings of Salman et al. [40], who have found that higher tensile strength and tensile modulus of plain fabric is associated with the differences in the load-distribution properties of the yarns along the longitudinal and transverse directions, which results in higher stress uptake capacity. In another study conducted by McDaniels et al. [41], tensile loading in fabrics induces transverse loads at warp-weft yarn overlap section (yarn interlacement) as crimped yarns tend to straighten. This reduces the translation of fibre strength to fabric strength and decreases long-term fatigue and creep rupture performance.

**Figure 5:**
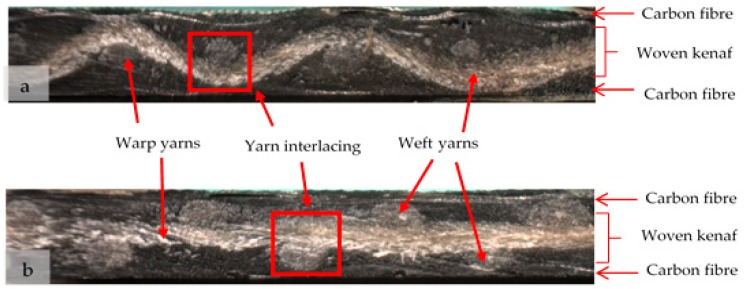
Cross section view of (**a**) warp direction and (**b**) weft view of laminated woven composite from plain fabric under optical microscope.

**Figure 6:**
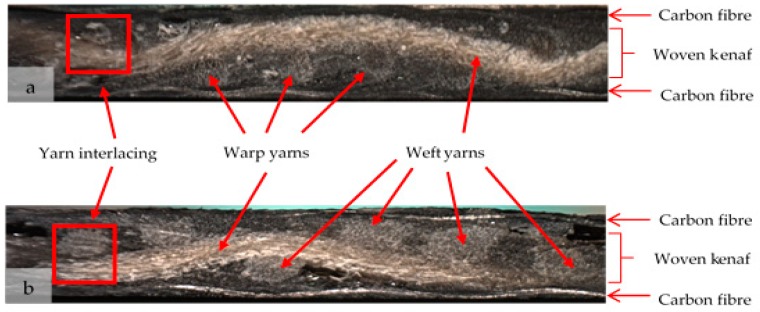
Cross section view of (**a**) warp direction and (**b**) weft view of laminated woven composite from satin fabric under optical microscope.

Figure 5 and Figure 6 show the cross section view of (a) warp direction and (b) weft view of laminated woven composite from plain fabric and satin fabric, respectively under optical microscope. The frequency of yarn interlacing and the linearity of the yarn segments distinguish both fabrics. The plain weave has the highest frequency of yarn interlacing, whereas the satin weave has the least number of yarn interlacing. Due to a more consistent and higher amount of yarn-to-yarn interlacement in plain-designed composite, the applied stress is distributed uniformly and the cracks ran transversally in all direction, perpendicular to the loading direction due to isotropic woven packing. Therefore, it can withstand greater tension that hold to each other, stress transfer gradually to the adjacent yarn and results in less slippage in the structure. Nonetheless, in the satin-designed composite, there is anisotropic arrangement of yarns in the packing structure, thus, lacking uniform distribution of force along the applied stress to support the transference of load. Hence, cracks and damages are easily formed. In addition, there are higher numbers of floating yarn in satin arrangement that cooperate with loose satin structure. This led to stress being transferred intermittently to neighbouring yarn and consequently reducing the strength because failure could easily have occurred at this zone and disseminated to the adjacent yarns.

**Figure 7:**
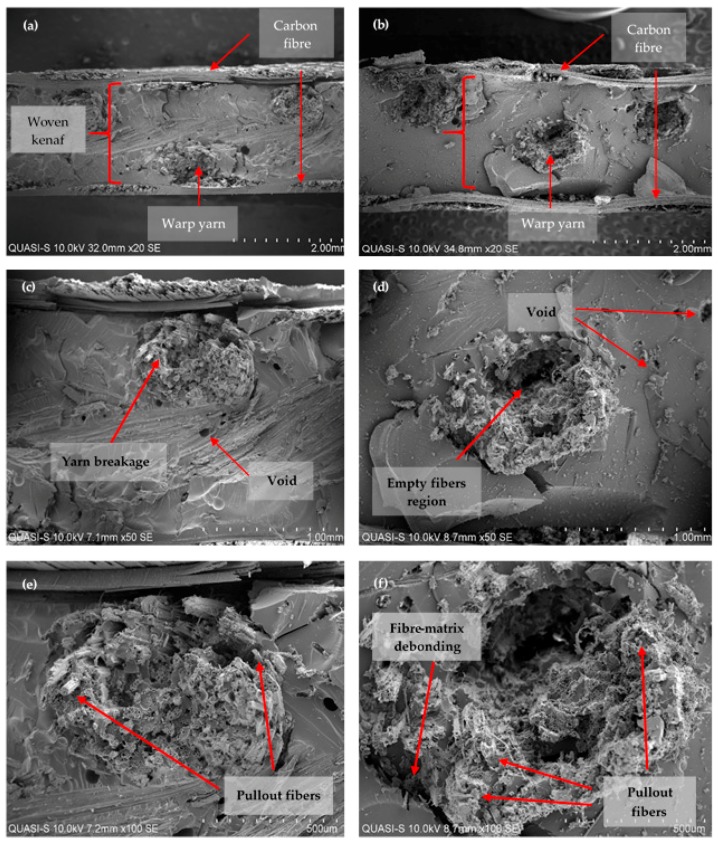
The SEM micrograph of tensile fracture surface of laminated woven kenaf composite: (**a**) fracture surface at low magnification of plain-designed composite and (**b**) satin-designed composite; (**c**) yarn fracture in plain-designed composite and (**d**) yarn fracture in satin-designed composite; (**e**) fibre pullout in plain-designed composite at 100× magnification and (**f**) fibre-matrix debonding and pullout in satin-designed composite at 100× magnification.

The morphology of the tensile tested composites of the woven kenaf/carbon fibre hybrid composite for plain and satin fabric are shown in Figure 7. Figure 7a,b show the cross-sectional view of the fractured surface from the tensile failure test for plain and satin woven composite, respectively. More severe broken yarns were observed on the failed specimens of the satin-designed composite compared to those of the plain-designed composite. The latter appears to have better bonding (Figure 7c) as is shown by the presence where most parts of the failed fibres are still in aggregates form except for a small number of individual pull-out fibres. Satin-designed composite seems to experience serious interfacial debonding particularly at the fibre bundles of the kenaf yarns (Figure 7d). It is noted that empty fibres regions (i.e., voids) are also present which are caused by the tensile force under tension loading. High magnification of SEM images of the failure as determined by the fractured surface confirmed that the mode of failure was due to either fibre fracture, pull-out fibres and voids and their combinations which had resulted from different fibre structure in the core layer of the woven kenaf/carbon fibre hybrid composite (Figure 7e,f) According to Zhou et al. [42], the interfacial debonding and matrix failure are closely associated to the interlaced constitution of the woven fabrics. Additionally, the tensile stresses from tensile load shifting from matrix to yarns are dependent upon optimum stresses that can overcome the friction resistance. The weak bonding strength of epoxy-yarn makes the fibre bundles break and pulls out from the matrix. 

Several researchers had highlighted that the fibre volume fraction and void content of composite influenced the mechanical properties particularly tensile properties [35,43,44]. Junior et al. [45] found that tensile behaviour of ramie/cotton polyester composite was governed mainly by volume fraction, rather than by yarn size and fabric compactness. On the other hand, Zhu et al. [46] reported that tensile strength of carbon-epoxy laminated composite were reduced as the void content increased because the void introduced initiation and formation of the cracks in composite structure. Hernandez et al. [47] found the increasing of void content was related to the result of air trap and wrinkles created during lay-up process. 

#### 3.2.2. Flexural Strength

The ANOVA in Table 5 also found that flexural strength was not affected by both weave design and fabric count. However, flexural modulus was significantly affected by the weave design. Upon further analysis using LSD (Table 7), the plain design appears to give a significantly higher flexural modulus. This result suggests that by changing the fabric type from satin to plain, the flexural modulus of the composite would improve by 23%.

**Figure 8:**
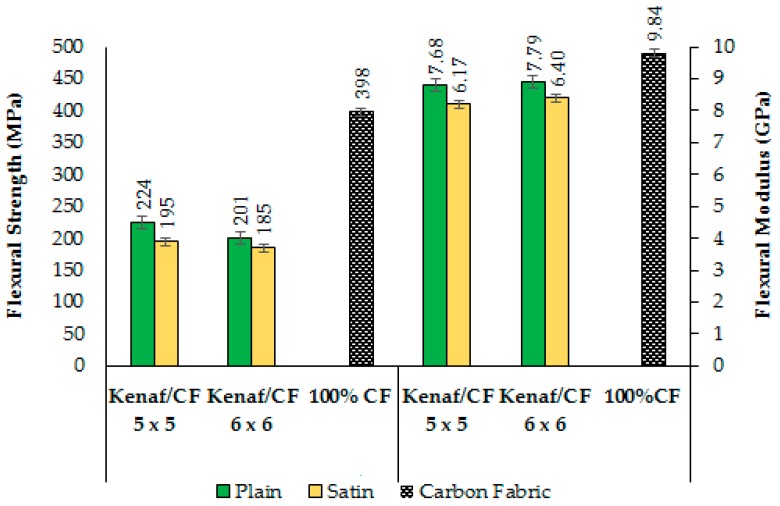
Flexural strength and flexural modulus of woven kenaf/carbon fibre hybrid composite.

Comparison of the composite flexural properties as affected by different combination of fabric count and weave design are shown in Figure 8. From the figure, CP5 exhibited the highest flexural strength (224.33 MPa) and CP6 exhibited the highest flexural modulus (7.79 GPa), while CS6 showed the lowest flexural strength (185.04 MPa) and CS5 showed the lowest flexural modulus (6.17 GPa). 

All the hybrid composites have a significantly lower flexural strength than the 100% carbon fibre laminates. This is expected as carbon fibre can transfer and withstand flexural load more efficiently because the strength and rigidity of a carbon fibre component have been created by positioning fabrics in a specific way. The relatively higher flexural strength for composite made from plain fabric was largely attributed to the interlocking structure of plain fabric. In plain fabric, the warp and weft yarn are aligned and formed a criss-cross arrangement. This type of yarn arrangement prevents any extension of the yarn along the load directions, which increases the bending load capacity and results in better composite strength properties. The effect of fibre orientation on the flexural modulus has been reported by many studies [16,37,48,49]. Fibre orientation affects the flexural properties by influencing the whole yarn structure, yielding an improvement in fabric orientation. This better fabric arrangement can explain the increased in flexural strength of woven composite that are made from plain-weave fabric

**Figure 9:**
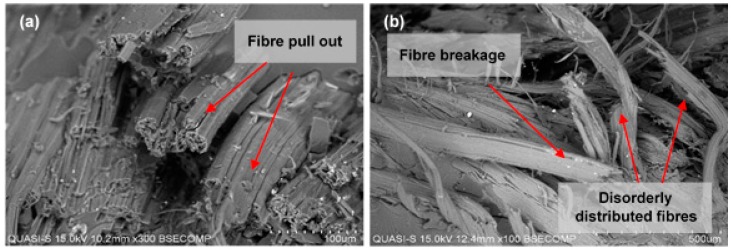
The SEM micrograph images of the flexural failure surfaces of woven kenaf/carbon fibre hybrid composite: **(a**) fibre breakage and (**b**) fibre pull-outs and disorder arrangement of fiber on the tested specimens.

On the contrary, in satin-weave fabric, there is a complex arrangement of warp and weft yarns, which allows longer float yarns across the warp/weft yarns. The less stable arrangement in satin fabric obstructs distribution of this load, thus giving low flexural strength, as is shown by the fibre breakage and pull-outs in Figure 9. In addition, plain weave fabric has better wetting properties since it contains large amount of fibres and higher kinetics rate of water absorption [50,51]. The good wetting properties give rise to resin penetration and subsequently produced composite with better strength properties. 

In the case of flexural modulus, as expected the values that were obtained from samples with 100% carbon fibre was higher than those obtained from the samples of hybrid with woven kenaf. However, it was observed that woven kenaf/carbon fibre hybrid composite have comparable flexural modulus values when compared to pure carbon fibre composite. A significant increase of the flexural modulus when woven kenaf of 6 × 6 fabric count are used, both for composites with plain and satin design, has been observed when compared to composite with 5 × 5 of fabric count. In the three-point flexural test, a vertical load is applied, the compression load associated with the deformation is generated on that upper side, whereas on the opposite side, a tensile load is generated, leading to a tensile deformation of test specimens. Since the sample experiences both the compressive and tensile forces during a flexural test, this may explain the greater sensitivity of the flexural data to this phenomenon.

According to Dhakal et al. [31], in the flexural test, surface of the composite is subjected to higher compression stress at the core part. Therefore, the flexural modulus is controlled by the strength of the intense reinforcement, i.e., the woven kenaf in this study. The woven kenaf stiffness is apparently dependent on fabric arrangements, such increment of the modulus with the fabric structure implied a good dispersion of the reinforcements. The function of woven kenaf as a rigid filler was assumed to have enhanced the stiffness of polymer matrix and its strong interaction with epoxy matrix [52]. This implies that the stronger carbon fibres in the outer layers might have influenced the flexural strength of the hybrid woven composites. Accordingly, the woven kenaf plays a major role in increasing the stiffness of hybrid woven composites by offsetting the low elongation of woven kenaf.

**Figure 10:**
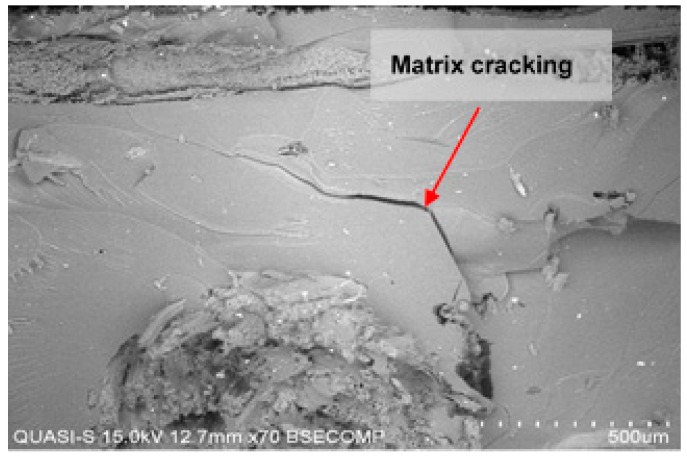
Matrix cracking is observed at resin rich area in 6 × 6 fabric.

Even though fabric count has no significant effects on both flexural strength and flexural modulus, it is interesting to note that fabric count of 5 × 5 has consistently produced a higher flexural strength and modulus. This may be due to the effectiveness of epoxy resin in the 5 × 5 fabric that is spread into woven kenaf and carbon fibre, hence, enhancing kenaf–carbon bonding adhesion. Lee et al. [53] have concluded that flexural modulus is dominantly affected by the effectiveness of epoxy resin in the reinforcement materials. In the 5 × 5 fabric, less fabric tightness was found than in the 6 × 6 fabric due to less yarn numbers, hence a higher mobility and wetting properties within the fabric structure. It appears that the higher porosity in the 5 × 5 fabric provides better penetration of epoxy resin. Conversely, the higher tightness in the 6 × 6 fabric provides poor resin penetration resulting in composites of low flexural strength. This is due to the presence of ‘resin-rich-area’ as more resin is being accumulated on the surface instead of penetrating into the next layer. This area is the weakest point where poor bonding between fabric and epoxy creates cracking, propagating through the epoxy matrix (Figure 10) causing decohesion and separation of fibrils and consequently reduced its strength.

#### 3.2.3. Impact Strength

**Figure 11:**
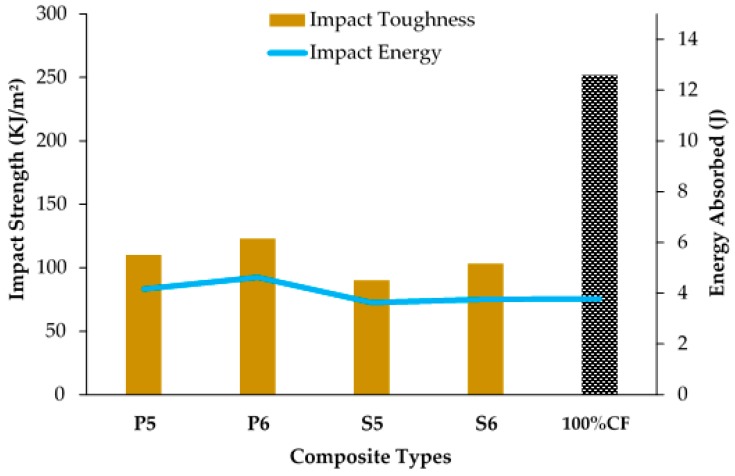
Impact strength and total energy of woven kenaf/carbon fibre hybrid composite and carbon fibre composite.

Figure 11 presents the impact strength and impact energy of woven kenaf/carbon fibre hybrid composite. All of the hybrid composites CP5, CP6, CS5, and CS6 has low impact energy compares to 100% carbon fibre laminated composite, CF. In contrast, the energy that has been absorbed by hybrid woven composite exhibits comparable values to carbon fibre laminated composite. Joseph et al. [54] have mentioned that, the impact strength of composite is driven by several factors, including the types of polymers, fibre-matrix interface, structure and arrangement of materials that are used for composite. 

**Figure 12:**
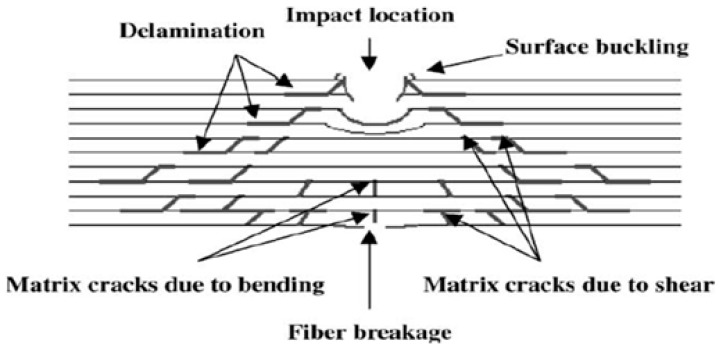
Schematic representation showing a typical impact damage mode for a composite laminate [58].

Based on the results, the impact strength and energy absorbed by sample CP6 indicated the highest values at 123.07 kJ/m² and 4.62 J, respectively. Weave design plays an important role as plain fabric in the composite has a higher energy absorption capacity due to its interlocking structure that contributes to composite strength [55]. In addition, plain fabric also has high elongation capabilities which leads to high impact strength compares to satin fabric. The tightening effect of plain fabric has increased the specimen stiffness. Stiffer materials deform less and carry higher load and increase its ability to absorb impacts [56]. When the impact load is applied on the specimen, the upper layer is under compression stress while the lower layer is under tension stress. The middle layer is put in shear stress. The woven fabrics structure parameters in the middle layer affect the resistance behavior during load. Throughout the impact load, cracks start at the impact side and spread into the loading direction. Figure 12 shows the typical impact damage mode in composite laminate. The middle layer helps in absorbing a large amount of impact energy. The firm structure of plain fabric offers an obstacle to the spread of further cracking by absorbing and disseminating the impact stress before failure. Conversely, satin fabric contains more floating yarn that can bring to the yarn slip phenomena [57]. This phenomena happens in satin fabric because of the lesser number of interlacements between warp and weft yarn. As a result, some yarns are not held firmly in the satin structure and result in low absorbing impact energy. Fibre breakage and fibre pull out occurred due to maximum energy absorption which lead to delamination.

Hybrid composite with woven kenaf resulted in better energy absorbed, particularly in the case of CP6 that showed significant increment (22.47%) when plain fabric with 6 × 6 of fabric count was used as the core in the composite. It was also found that hybrid with kenaf plain-designed composite absorbed more energy than satin-designed composite, and was slightly higher than carbon fibre composite. This finding is in agreement with the research work done by Wambua et al. [59] that attained better energy absorption by incorporating natural fibres (flax, hemp, and jute) in the woven form to the middle layer of natural fibres/mild steel hybrids composite. This is related to the unique energy absorption properties by natural fibres that have acted as a stiff layer to deflect and absorb more impact energy, as compared to carbon fibre that is more brittle. Furthermore, fibres that are derived from plants have low level of embodied energy than synthetic fibres and contain cellulose as their major structural component. High cellulose content and cellulose microfibrils which are aligned in the fibre direction give higher performance in energy absorbtion apart from having higher specific Young’s modulus and tensile strengths than synthetic fibres [60]. 

**Figure 13:**
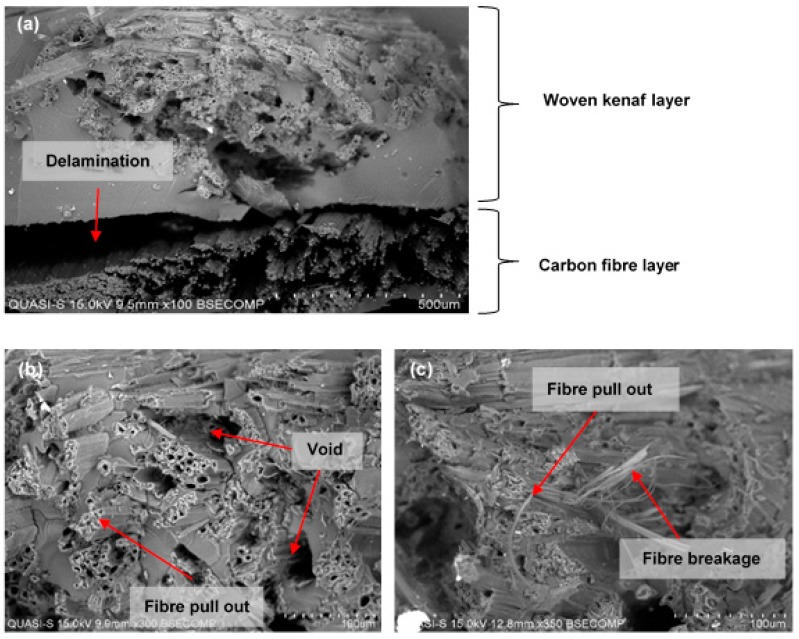
The SEM micrograph of impact fracture surface of laminated satin woven kenaf composite. (**a**) Delamination between layers; (**b**,**c**) fibre pull out, breakage and voids can be observed at higher magnifications in satin-designed composite.

Based on the morphology of the impact fractured surface in Figure 13, it was observed that the composites failed by a combination of intense fibre pull out, fibre breakage, delamination between layers and voids in the composite. These failure modes occurred more in the satin fabric specimen. Some of these failure modes were also observed in the plain fabric samples, but the extent of the failures differed from that of the satin fabric sample. Many researchers [61,62,63] had mentioned that the weave design was responsible for determining the impact toughness of the composite. Carbon fibre and epoxy matrix were sheared and delaminated as shown in the Figure 13a. A crack through the woven kenaf/carbon fibre and epoxy interface after the failure can be seen clearly, indicating that the phenomenon of fibre pull-out happened to a large degree (Figure 13b,c). This failure mode is in agreement with Aly et al. [61] who have concluded that the impact properties are strongly affected by woven fabric structure and the resin properties. The failure mechanisms described above were also observed in this study. Another factor for causing these phenomenon might be related to the plain fabric’s high cover factor and porosity values. Therefore, plain fabrics are better in interfacial adhesion which leads to good resin-fabrics penetration. Thus, the composites experienced less kenaf fibre pull out and void in the composite, resulting in high impact resistance. Pickering et al. [60] have reported that the impact absorption capability of composite material depends upon the interfacial strength between the fibres and the matrix. These findings were also supported by Salman et al. [40] who have stated that the plain fabric could add structural strength and leads to an increase in strength as well as energy absorption capacity of the composite.

It was particularly noticeable that the impact properties increased with an increase in fabric count to 6 × 6. This indicates that the addition of fibre content in the composites has increased energy absorption capacity or makes the composite to be more resistance to impact stress. This can be interpreted that increasing the number of warp yarn has increased the numbers of yarns that are able to bear the impact load. Impact stress can be distributed efficiently in the composite with higher amount of fibres and delay delamination [64]. Closer weave structure improves energy absorption because high fabric density slows down crack growth and results in a smaller damage length [22]. Moreover, woven composite with high fabric density has higher impact and damage tolerance due to reduced impact damage which is a result from the higher number of yarn interlacement in a preform [65]. Hosur et al. [66] observed that the impact response of plain fabric composites reduced the delamination initiation due to fibre interlacement in their structure. They also indicated that the bottom layer of the woven laminates did not split during impact loading. Plain fabric composite also have better impact resistance due to the higher transverse strength in woven composites that are created by the interlacement of the weft and warp yarns in the preform [67]. Furthermore, CP6 fabric and carbon fibre bond better with the epoxy resin, provide better adhesion between fabric and resin, thus less fibre pullout and create strong bonding. This bonding results in great amount of impact energy absorption.

## 4. Conclusions

The mechanical properties of woven kenaf/carbon fibre reinforced epoxy hybrid composites were affected by the weave designs and the fabric counts of woven kenaf, with weave design being significantly affected more by the mechanical properties. By using plain fabric, the tensile and impact strength were improved compared with satin fabric, and using 5 × 5 of fabric count had improved flexural modulus compared with 6 × 6. The tensile and impact strength of the composite at fabric count of 6 × 6 was found to be higher than other composites indicating that composites were strongly determined by the fabric structure, fabric strength and fibre content. Plain fabric and the 5 × 5 of fabric count showed higher flexural strength due to the better adhesion of woven kenaf in the epoxy resin compared to satin fabric and the 5 × 5 of fabric count. SEM examinations of failure test specimens revealed poor adhesion in the composite structure and the failures were caused by fibre pullout, fibre-resin debonding, and some voids. The increment in the fibre volume fraction and the reduction in void content had increased the tensile strength of the composite simultaneously. The produced hybrid woven composites could potentially be used for military applications such as the production of body armour and a ballistic helmet. Other applications include automotive and construction industries.

## Figures and Tables

**Table 1:** Some reported studies on woven natural fibre reinforced polymer composites.

Fibre Types	Matrix Type	References
Woven jute and glass fibre	Polyester (PE)	Ahmed et al. [8]
Woven flax	Epoxy	Liu and Huges [20]
Woven coir and Kevlar	Epoxy	Azrin Hani et al. [22]
Woven kenaf fibre	Glass, nylon fibre	Me et al. [13]
Plain and twill hemp fabric	Polylactic acid (PLA)	Song et al. [4]
Woven banana and woven kenaf	Polyester (PE)	Alavudeen et al. [18]
Hemp woven fabric	Polylactic acid (PLA)	Baghaei et al. [23]
Woven kenaf and Kevlar fabric	Epoxy	Yahaya et al. [24]
Woven jute	Epoxy	Abdellaoui et al. [6]
Jute fabric	Poly(l-lactic acid) (PLLA)	Khan et al. [5]
Woven kenaf and non-woven mat bamboo	Epoxy	Chee et al. [25]

**Table 2:** Properties of carbon fibre supplied by Spinteks Tekstil Ins.

Properties	Carbon Fibre
Density	1.78 g/m^3^
Thickness	0.20 mm
Tensile Strength	3800 MPa
Tensile Modulus	240 GPa
Strain	1.6%

**Table 3:** Composition of kenaf woven in the hybrid composite.

Code	Weave Design	Fabric Count
CP5	Plain	5 × 5
CP6	Plain	6 × 6
CS5	Satin	5 × 5
CS6	Satin	6 × 6

**Table 4:** Composite density and volumetric composition of woven kenaf/carbon fibre hybrid composite.

Type of Composite	Density (g/cm^3^)	Woven Kenaf		Carbon Fibre		Total Fibre Vol. Frac. *V_f_* (%)	Void Vol. Frac. (%)
		Wt. Frac.	Vol. Frac.	Wt. Frac.	Vol. Frac.		
		Wkf (%)	Vkf (%)	Wcf (%)	Vcf (%)		
CP5	0.98	20.37	17.45	11.27	12.27	28.62	0.32
CP6	1.19	20.95	16.33	11.33	12.31	29.74	0.38
CS5	1.11	21.98	20.14	11.45	12.23	31.11	0.62
CS6	1.24	22.60	18.89	11.85	12.25	32.33	0.75

**Table 5:** Summary of ANOVA on the mechanical properties of woven kenaf/carbon fibre hybrid composite.

Variables	df	*p*-Value					
		Tensile Strength	Tensile Modulus	Flexural Strength	Flexural Modulus	Impact Strength	Impact Energy
Weave Design (WD)	2	0.0002 ***	0.2526 ^ns^	0.5636 ^ns^	0.0232 **	0.0227 **	0.0422 **
Fabric Count (FC)	2	0.1430 ^ns^	0.1182 ^ns^	0.1675 ^ns^	0.7831 ^ns^	0.1174 ^ns^	0.3522 ^ns^
Interaction (WD × FC)	4	0.2546 ^ns^	0.2338 ^ns^	0.0661 *	0.9206 ^ns^	0.9825 ^ns^	0.6036 ^ns^

df: degree of freedom; ***: Significantly different at *p* ≤ 0.01; **: Significantly different at *p* ≤ 0.05; *: Significantly different at *p* ≤ 0.10; ns: not significant.

**Table 6:** Effect of kenaf weave design on the tensile strength of woven kenaf/carbon fibre hybrid composite.

Weave Design	Tensile Strength (MPa)
Plain	122.04 ^a^
Satin	104.96 ^b^
LSD	8.3841

Note: Means followed by the same letters a, b, are not significantly different at *p* ≤ 0.05 according to Least Significant Difference (LSD) method.

**Table 7:** Flexural properties of woven kenaf/carbon fibre hybrid composite at different weave design.

Weave Design	Flexural Modulus (GPa)
Plain	7.74 ^a^
Satin	6.28 ^b^
LSD	1.209

Note: Means followed by the same letters a, b, are not significantly different at *p* ≤ 0.05 according to Least Significant Difference (LSD) method.
